# Results of an Eight-Year Extraction of Phosphorus Minerals within the Seymchan Meteorite

**DOI:** 10.3390/life12101591

**Published:** 2022-10-12

**Authors:** Maheen Gull, Tian Feng, Matthew A. Pasek

**Affiliations:** School of Geosciences, University of South Florida, Tampa, FL 33584, USA

**Keywords:** phosphorus, early Earth, origin of life, meteorites, phosphides, phosphite

## Abstract

**Simple Summary:**

Phosphorus (P) is essential to life in the form of various phosphate esters that make up DNA, RNA and other cellular structures. It mainly exists in the form of phosphate. However, other reduced-oxidation-state P compounds have also been found in natural waters, in living organisms, and in natural glasses formed by lightning called fulgurites. Iron meteorites often bear a P mineral, schreibersite, which corrodes in water to produce various P species including reduced-oxidation-state compounds such as phosphite. This form of natural P is reactive and unstable as ultimately the phosphides and phosphites convert into phosphate, the most stable form of P on an oxidizing world. Previous analyses of 3.5 billion rocks identified various P species including phosphite revealing that phosphite was present on the early Earth and, despite its reactivity, it was stable under geologic timescales. In the present communication, we present the analyses of a meteoritic sample, the pallasite Seymchan, which is rich in the mineral schreibersite and was allowed to corrode for eight years in water. At room temperature, the schreibersite corroded in water to give P species including phosphate and phosphite. These results indicate that phosphite is not an ephemeral species and it is stable enough to be detected in multiple environments.

**Abstract:**

In-fall of extraterrestrial material including meteorites and interstellar dust particles during the late heavy bombardment are known to have brought substantial amounts of reduced oxidation-state phosphorus to the early Earth in the form of siderophilic minerals, e.g., schreibersite ((FeNi)_3_P). In this report, we present results on the reaction of meteoritic phosphide minerals in the Seymchan meteorite in ultrapure water for 8 years. The ions produced during schreibersite corrosion (phosphite, hypophosphate, pyrophosphate, and phosphate) are stable and persistent in aqueous solution over this timescale. These results were also compared with the short-term corrosion reactions of the meteoritic mineral schreibersite’s synthetic analog Fe_3_P in aqueous and non-aqueous solutions (ultrapure water and formamide). This finding suggests that the reduced-oxidation-state phosphorus (P) compounds including phosphite could be ubiquitous and stable on the early Earth over a long span of time and such compounds could be readily available on the early Earth.

## 1. Introduction

Meteoritic impacts have been directly linked with the origin of life on the early Earth [1,2]. Since the identification of abundant organics in the Murchison meteorite, prebiotic chemists have assumed that meteorites supplied extraterrestrial compounds to the early Earth, which could have kick-started the events of prebiotic syntheses [3,4,5,6,7,8,9,10,11,12,13]. In addition to organic compounds, meteorites are also believed to have supplied a non-negligible portion of the phosphorus within the Earth’s crust, possibly through a heavy bombardment [14,15]. Studies have shown that meteoritic mineral schreibersite [(FeNi)_3_P]) is ubiquitous in iron meteorites and is common to many other meteorite classes [16,17]. This iron-nickel phosphide is known to be among the first inorganic P compounds to condense from the solar nebula as part of homogeneous accretion model and therefore, is considered to be one of the most ancient P minerals within our solar system [18].

Around 5–10% of all crustal P was at some stage delivered as meteoritic phosphide minerals [14,15,17]. Schreibersite is known to corrode in water by oxidation to release several inorganic P species in aqueous solution. These species include phosphite (HPO_3_^2−^ with P (III)), orthophosphate (HPO_4_^2−^ with P (V)), hypophosphate (HP_2_O_6_^3−^ with P (IV)), and pyrophosphate (HP_2_O_7_^3−^ with P (V)), respectively [11,15], with a concomitant release of H_2_.

Schreibersite or its corrosion products can react with organic compounds to form C-O-P and C-P type compounds [18,19,20,21,22]. These reactions established the case for schreibersite as a prebiotically relevant P containing mineral. Since around 70% of the Earth’s surface is covered with water, much of this meteoritic material would have hit oceans [23], and schreibersite would have corroded within water. The discovery of phosphite signatures in the ancient marine carbonates [20] supports the possibility that the corrosion products of schreibersite may have been present in Archean oceans. However, it is not known that the phosphite ions are persistent and can be stable for a long timespan.

In the present study, we describe the corrosion of schreibersite within the meteorite Seymchan (a pallasite that is compositionally similar to IIE irons [24], and which is relatively enriched in nickel at ~9 wt.%) that was left to corrode in deionized water for 8 years. Seymchan bears a few percent by volume of schreibersite [16]. This work specifically identifies whether the ions generated by schreibersite are ephemeral, or whether they can persist for years.

Furthermore, the corrosion reaction (P) products from the Seymchan meteorites were also compared with that of the synthetic analog Fe_3_P. The corrosion reactions of Fe_3_P were carried out at the room temperature (25 °C) for shorter timescales. Two types of solvents were employed; (1) ultrapure water as an aqueous medium, and (2) formamide as a non-aqueous medium. The latter option was chosen because of its availability and ubiquity in the interstellar regions and various habitable zones in our galaxy [25,26]. Furthermore, it is also considered to be a prebiotically relevant alternative solvent due to its remarkable solvation capabilities and ability to provide an anhydrous medium for phosphorylation and condensation reactions [27,28,29,30,31].

## 2. Materials and Methods

Both iron phosphide or Fe_3_P (99.5% trace metals basis) and formamide (from BioUltra) for molecular biology, ≥99.5% were purchased from Sigma Aldrich (St. Louis, MO, USA). The doubly deionized water (DDI) was produced in house using a Barnstead (Dubuque, IA, USA) NANO pure^®^ Diamond Analytical combined reverse osmosis-deionization system as reported previously [20,21,22]. It should be noted that the authentic meteoritic mineral schreibersite [(Fe,Ni)_3_P] or its commercially available synthetic analogue (Fe_3_P) upon corrosion release same P species as reported previously [15,16,17,18,19,20,21,22], but this study further investigates the use of authentic schreibersite in corrosion experiments.

The Seymchan meteorite sample was procured from meteorite dealers in the form of shavings covered with oil—done by the dealers during the preparation of Seymchan into slices to sell to collectors—which prevented oxidation and corrosion of these samples in the air. The oil was removed by placing the shavings into a clean flask containing acetone, sealing the container, and stirring the solution on a stir plate with the help of magnetic stirrer. The mixture was allowed to stir overnight to ensure dissolution of oil into the acetone. The shavings were then filtered and rinsed off with acetone. The process was repeated 2 times and after that was allowed to completely air dry and followed by heating in the oven at 50 °C for 5–6 h to ensure evaporation of the organic solvent. The mean surface area of the Seymchan was around 0.001 m^2^/g based on the dimensions of the shavings (individually ~5 mm × ~1 mm × ~0.3 mm).

About 5 g Seymchan was added to 15 mL DDI (or ultrapure) water with no other additives and was sealed tightly in a glass vial under air and was set to corrode at room temperature for about 8 years. The glass vial was placed in a completely dark cabinet. No water evaporated from this vial over those 8 years, and the material was not stirred over this time. For comparing the corrosion reaction P products of meteoritic phosphide from Seymchan with that of Fe_3_P, the following general procedure was followed. About 0.5 g Fe_3_P (to each reaction sample) was added to a clean, small glass vial containing 5 mL DDI (or ultrapure) water (labeled as sample B) and to a glass vial containing 5 mL of pure formamide (labeled as sample F). A clean magnetic stirrer was added to each glass vial containing the reaction mixture. The glass vials of the solution mixtures were then tightly sealed and were allowed to stir at the room temperature for about 2 weeks.

The release of P from the Seymchan and the phosphide corrosion reactions in the ultrapure water and formamide were studied by ^31^P-NMR (Varian, Palo Alto, CA, USA) as reported previously [32]. In case of Seymchan, the tightly sealed sample tube was opened after 8 years and to it 0.5 M Na_4_EDTA solution was added until the pH reached ~12. This step was done to help with the extraction of P species from the meteorite sample as previously [15,16,17,18,19,20,21,22,32]. The mixture containing meteorite shavings in DI water and EDTA solution was transferred to a clean glass vial to let it stir at room temperature for 1 day, followed by filtration of the solution, and drying at the room temperature. Similarly, prior to ^31^P-NMR analysis, both reaction samples B and F were also treated with 0.5 M Na_4_EDTA solution until the pH reached to be around 11–12. The solution changed color to blackish brown. It was filtered and about 7 mL of the filtrate was transferred to a clean watch glass and was allowed to dry at the room temperature. The dried mixture was rehydrated with D_2_O and was added to NMR tubes for the analysis. The dried mixture was rehydrated with 1 mL D_2_O and was analyzed by the NMR. The molarity [M] of the solution was calculated by the formula (Equation (1)) as suggested by [19].

(1) $$[M]=0.0075\;\times \;{\left(\frac{\left(\frac{S}{N}\;\right)}{\sqrt{\mathrm{Scans}}}\right)}^{2}+0.0007\;\times \;\left(\frac{\left(\frac{S}{N}\;\right)}{\sqrt{Scans}}\right)+0.0001$$

where S/N is the signal to noise ratio, and Scans is the number of NMR scans taken. As reported in the previous studies [19]. this relationship was empirically determined and is accurate to about 10% over the range of 10^−4^ to 10^−2^ M based on the sample spectra obtained [19].

## 3. Results

**Figure 1:**
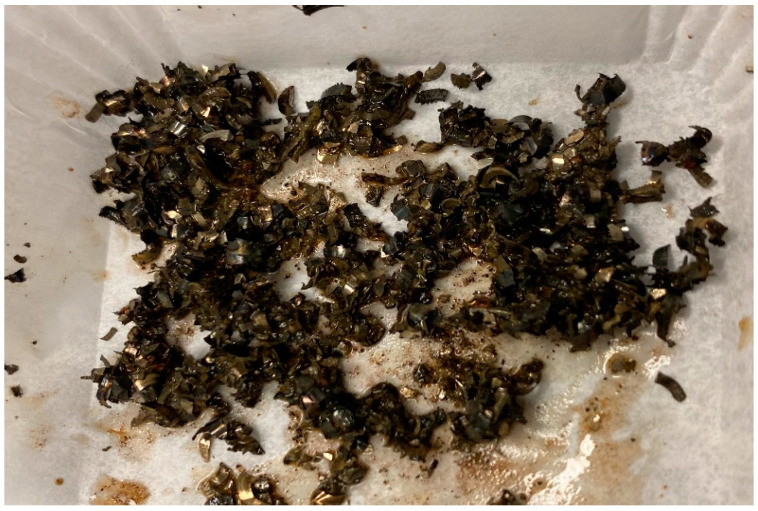
Seymchan meteorite (shavings) post 8 years corrosion reaction at room temperature.

The phosphide from the meteorite sample leached into the solution as various forms of P including orthophosphate, phosphite, pyrophosphate and hypophosphate. In addition, after being immersed in water the meteoritic shavings appeared to be somewhat rusty but all in all still retained the metallic luster of the meteoritic shavings (Figure 1). Interestingly, a few of the corroded shavings were brought closer to the magnet and were indeed pulled by the magnet, implying that even after 8 years the shavings were still not completely oxidized.

**Figure 2:**
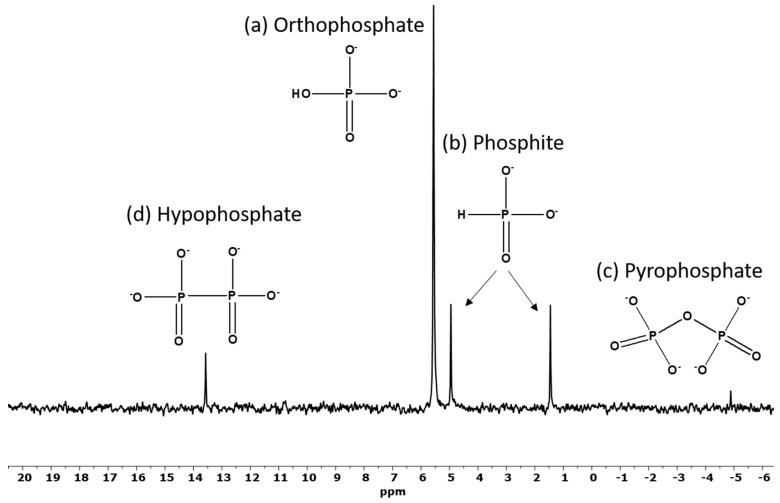
Corrosion reaction of Seymchan meteorite (sample SEY) stored at room temperature for 8 years. The *Y*-axis is in intensity and has arbitrary units.

The ^31^P-NMR studies of the meteorite extract solutions (Figure 2, Table 1) identified four major inorganic P species including: orthophosphate, phosphite, pyrophosphate and hypophosphate. The concentrations of the P species in the Seymchan solution were determined by the integration of the ^31^P-NMR spectra. It is important to mention here that NMR integration can be quantitative if the integrations are done over a narrow range of frequencies, e.g., less than 50 ppm [19]. In the proton-coupled mode of the NMR, the phosphite peak showed the typical split into a doublet which was identified by measuring the coupling constant (around 570 Hz) [33]. The measuring of the coupling constant distinguished phosphite from the orthophosphate peak which also located around the same region (~5.5 ppm). The pyrophosphate peak was located at −4.8 ppm and hypophosphate peak was identified to be around 13.5 ppm [19]. ^31^P-NMR spectrum is shown in the H-coupled mode of ^31^P-NMR.

**Figure 3:**
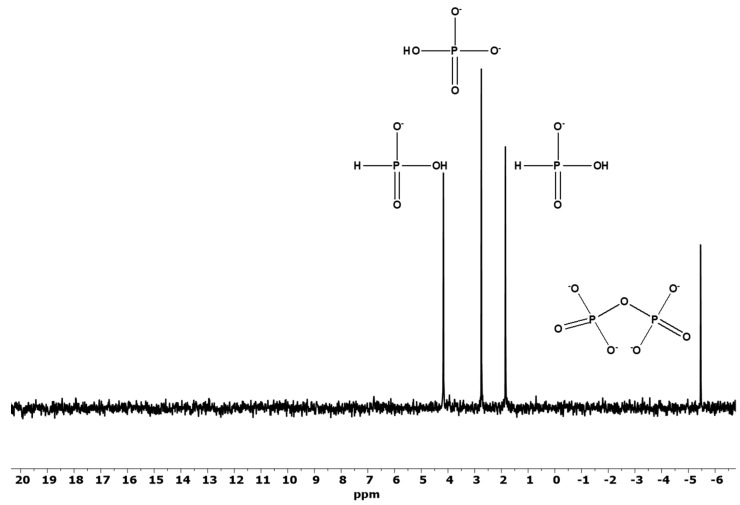
Corrosion reactions of Fe_3_P in pure formamide (sample F). The *Y*-axis is in intensity and has arbitrary units.

**Figure 4:**
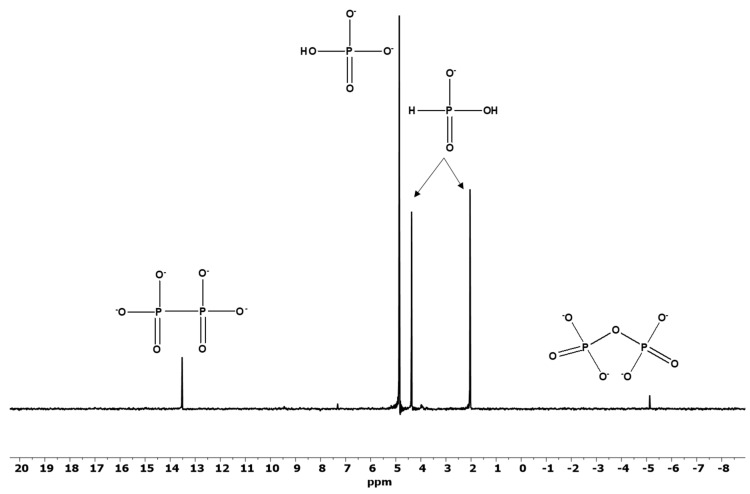
Corrosion reaction of Fe_3_P in ultrapure water (sample B). The *Y*-axis is in intensity and has arbitrary units.

The ^31^P-NMR studies of the corrosion reaction of Fe_3_P in the formamide (sample F, Table 1) and in the ultrapure water (sample B, Table 1) revealed similar reaction products with quantitatively different ratios as observed in the meteorite Seymchan’s solution extract and as also reported previously [19]. The major species in all samples included orthophosphate (HPO_4_)^2−^, phosphite (HPO_3_)^2−^, pyrophosphate (P_2_O_7_)^4−^ and hypophosphate (P_2_O_6_)^4−^, respectively. Interestingly, the highest yield of the pyrophosphate (condensed phosphates) was observed when formamide was used as a solvent (sample F) (Figure 3). This reaction sample also showed the highest amount of phosphite indicating that the oxidation of the P in non-aqueous media proceeds at rather slower rates as compared in the aqueous solutions. No hypophosphate was observed in sample F further indicating a relatively slower rate of corrosion and possibly the free radicals to form this P species were not generated as effectively in the formamide medium. The overall higher corrosion rate was observed in sample B (Figure 4) containing ultrapure water (or doubly de-ionized water) as a solvent thus indicating the ease of corrosion under aqueous conditions, also accompanied with facile oxidation. Furthermore, the highest concentration of P products was in sample B (Table 1).

## 4. Discussion

After 8 years, the ^31^P-NMR results demonstrated that the P compounds within the Seymchan–water solution were comparable to that of a typical ^31^P-NMR spectrum of schreibersite’s analog Fe_3_P (samples B and F). Phosphite—typically considered to be highly reactive—was present even after 8 years of corrosion under air, suggesting the phosphite species is stable even though this compound is out of equilibrium and should have oxidized by reaction with water to form phosphate [19,34]. These results are also consistent with the results of the hydrothermal treatment of the Seymchan meteorite [24] that studied the schreibersite corrosion from Seymchan after ~2 weeks. The results showed that the treatment of Seymchan shavings afforded various P species including hypophosphate (4% of solution P), orthophosphate (34%), phosphite (49%) and pyrophosphate (13%), respectively [24]. These results are comparable to the present study (Table 1), though phosphite is lower after 8 years.

It is intriguing that room temperature reactions of schreibersite present in the meteorite can afford the formation of condensed phosphates including pyrophosphate, and the rather unstable phosphite is still present (Figure 2). Previous studies have shown that in the presence of Fe metal, the rate of conversion of phosphite to phosphate in one day is about 46% under air [19]. Such reactions are also possible for the Seymchan meteorite as it is known to be rich in Fe. Furthermore, the kinetic stability of orthophosphate, hypophosphate and pyrophosphate has been found to be more than phosphite in the presence of oxidizing radicals [19,35].

Considering that the ancient oceans were anoxic and Fe (II)-rich [36,37], phosphate is known to adsorb to iron oxyhydroxides, which may have depleted early oceans with respect to total phosphate. The effect of iron oxyhydroxides on reduced P compounds is unknown, but Pasek and colleagues have shown that the reduced-oxidation-state P compounds including phosphite and hypophosphite oxidize in the presence of Fe (II) and H_2_O_2_ under prebiotic conditions. This process gives orthophosphate, pyrophosphate and triphosphate. This process occurs under oxic as well as anoxic conditions [38].

Alternatively, it has also been suggested that Archean oceans may have been strongly P-limited due to the selective binding of phosphate to iron oxyhydroxide [39,40,41]. Routes around this issue may have included plausible pathway of the reduction of phosphate to phopshite by iron (II) at low diagenetic temperatures (160–200 °C) and under a dinitrogen atmosphere. This suggests a plausible geochemical pathway of solubilizing P in the Archean ocean and indicates that the reduction of phosphate to phosphite would have been widespread in the Archean [39].

Therefore, phosphite, and not necessarily phosphate, may have been a major P source in the early anoxic oceans and would definitely have played a key role in the origin of P biochemistry. Iron and phosphate (even as calcium phosphates) could have been ubiquitous in many Archean sediments, as these have already been co-located in hydrothermal plumes off the southern East Pacific Rise [42]. Therefore, the alteration of phosphate into phosphite in the presence of Fe (II) via diagenesis would have been possible in the ancient oceans [39].

Furthermore, considering that our leaching experiment was performed under neutral pH conditions (i.e., using doubly deionized water with pH = 7), similar to the increased pH values (∼6.5 to 7.0) of the early Archean oceans as reported by Halevy and Bachan [43]. It should be noted that the increase in the pH could slightly decline the rates of the meteoritic phosphide corrosion; however, the P-speciation would remain the same [19].

Given the amounts of siderophilic P estimated to have impacted the early Earth during the late heavy bombardment [44], it is highly likely that highly water soluble and chemically reactive P species were readily available on the early Earth ready to kick-start the process of phosphorylation and phosphonylation of the organic compounds present on the early Earth.

Pasek and Lauretta [45] suggested that iron meteorites with schreibersite may have delivered that about 10^8^ kg/yr of meteoritic P to the surface of the Earth, would have been exposed to aqueous modification [45]. Ritson et al. [46] similarly propose 3 × 10^7^ kg/year delivered during the late accretion of meteorite material to the Earth. These models suggest that reduced P could have played a major role in delivering the reactive P to the early oceans and, even though these compounds are more reactive than phosphate, they can persist for longer timescales. These results also complement our previous finding of phosphite signatures in the marine carbonates [20], which would have been brought to the oceans possibly during late bombardment period as discussed above and would have played a major role in contributing to supplying the reduced form of P to the early Earth.

Furthermore, the successful corrosion of Fe_3_P in pure formamide show remarkable promise for anhydrous chemistry and possible phosphorylation of the organics with better yields. For about 40 years, this solvent has been utilized both as a reactant as well as a solvent for the prebiotic syntheses of biomolecules [27,28,29,30,31,47]. One of the major advantages of considering formamide as a solvent in the prebiotic chemistry is due, in part, to the fact that it supports condensation reactions that are required for the phosphate ester formation [28].

Many successful experiments have shown its viability as a solvent for phosphorylation reactions [27,28,29,30,31]. Yet, using phosphates as phosphorylating agents still presents a challenge that can be overcome using schreibersite or Fe_3_P. These results suggest that schreibersite readily corrodes in formamide and releases various P species as it would in water-based corrosion reactions. Future studies would help further solve the “problem of phosphorus chemistry” when schreibersite (or Fe_3_P) is used as a phosphorylating agent and formamide is the solvent. This route can plausibly be beneficial in two ways; (1) to release more soluble forms of P and (2) favorable anhydrous conditions necessary for phosphorylation.

Furthermore, prior work has shown that the reduced-oxidation-state P compounds are quite capable of reacting with biomolecules to form organic-P compounds essential for the cellular membrane structures and DNA/RNA-forming units (simple nucleotide units) [20,21,22]. Our previous findings of detecting phosphite signatures in 3.5 billion-year-old marine carbonates [20] and the present findings thus strongly suggest that the reduced-oxidation-state P compounds were not only readily available but were also quite stable on the early Earth. These reduced-oxidation-state P compounds would have essentially taken part in kick-starting the origin of life chemistry on the early Earth.

## Figures and Tables

**Table 1:** Yields ^1^ (%) and molarities of various P species released as a consequence of Fe_3_P corrosion in the solution.

Sample	Yields (%)				Concentration of Various P Species in Solutions (mmolar)				^2^ Total Molarity (mmolar)
	(HPO_4_)^2−^	(HPO_3_)^2−^	(P_2_O_7_)^4−^	(P_2_O_6_)^4−^	[(HPO_4_)^2−^]	[(HPO_3_)^2−^]	[(P_2_O_7_)^4−^]	[(P_2_O_6_)^4−^]	
SEY	71.12	23.40	0.20	5.27	1.20	0.40	0.05	0.10	1.75
F	29.24	54.39	16.37	ND	2.1	2.5	0.3	0.0	4.90
B	48.26	42.69	2.55	6.50	748	413.7	0.95	13.5	1176.1

^1^ The yields of the corrosion P products of the corrosion reactions were calculated on the basis of the total phosphorus dissolved and by the peak integration method as previously reported [19]. ^2^ The total molarity of the solution here is the sum of the individual molarities of each of the inorganic P species including (HPO_4_)^2−^, (HPO_3_)^2−^, (P_2_O_7_)^4−^, (P_2_O_6_)^4−^. The molarities are represented in millimoles. In addition, the labeling of the samples and the solutions descriptions are mentioned in Table 1.

## Data Availability

Not Applicable.

## References

[1] Cockell C.S. (2006). The origin and emergence of life under impact bombardment. Philos. Trans. R. Soc. Lond. B Biol. Sci..

[2] Osinski G.R., Cockell C.S., Pontefract A., Sapers H.M. (2020). The role of meteorite impacts in the origin of life. Astrobiology.

[3] Mason B. (1963). Organic matter from space. Sci. Am..

[4] Fegley B., Prinn R.G., Hartman H., Watkins G.H. (1986). Chemical effects of large impacts on the Earth’s primitive atmosphere. Nature.

[5] Oró J., Mills T. (1989). Chemical evolution of primitive solar system bodies. Adv. Space Res..

[6] Chyba C., Sagan C. (1992). Endogenous production, exogenous delivery and impact-shock synthesis of organic molecules: An inventory for the origins of life. Nature.

[7] Kobayashi K., Kasamatsu T., Kaneko T., Saito T., Chela-Flores J., Raulin F. (1998). Production of organic compounds in interstellar space. Exobiology: Matter, Energy, and Information in the Origin and Evolution of Life in the Universe.

[8] Pohorille A. (2002). From organic molecules in space to the origins of life and back. Adv. Space Res..

[9] Ehrenfreund P., Cami J. (2010). Cosmic carbon chemistry: From the interstellar medium to the early Earth. Cold Spring Harb. Perspect. Biol..

[10] Ehrenfreund P., Spaans M., Holm N.G. (2011). The evolution of organic matter in space. Philos. Trans. R. Soc. A Math. Phys. Eng. Sci..

[11] Kwok S. (2016). Complex organics in space from Solar System to distant galaxies. Astron. Astrophys. Rev..

[12] Nakano H., Hirakawa N., Matsubara Y., Yamashita S., Okuchi T., Asahina K., Tanaka R., Suzuki N., Naraoka H., Takano Y. (2020). Precometary organic matter: A hidden reservoir of water inside the snow line. Sci. Rep..

[13] Gull M., Pasek M.A. (2021). The role of glycerol and its derivatives in the biochemistry of living organisms, and their prebiotic origin and significance in the evolution of life. Catalysts.

[14] Macià E., Hernández M.V., Oró J. (1997). Primary sources of phosphorus and phosphates in chemical evolution. Orig. Life Evol. Biosph..

[15] Pasek M.A., Lauretta D.S. (2005). Aqueous corrosion of phosphide minerals from iron meteorites: A highly reactive source of prebiotic phosphorus on the surface of the early Earth. Astrobiology.

[16] Pirim C., Pasek M.A., Sokolov D.A., Sidorov A.N., Gann R.D., Orlando T.M. (2014). Investigation of schreibersite and intrinsic oxidation products from Sikhote-Alin, Seymchan, and Odessa meteorites and Fe_3_P and Fe_2_NiP synthetic surrogates. Geochim. Cosmochim. Acta.

[17] Bryant D.E., Kee T.P. (2006). Direct evidence for the availability of reactive, water soluble phosphorus on the early Earth. H-Phosphinic acid from the Nantan meteorite. Chem. Commun..

[18] Pasek M.A. (2008). Rethinking early Earth phosphorus geochemistry. Proc. Natl. Acad. Sci. USA.

[19] Pasek M.A., Dworkin J., Lauretta D.S. (2007). A radical pathway for phosphorylation during schreibersite corrosion with implications for the origin of life. Geochim. Cosmochim. Acta.

[20] Pasek M.A., Harnmeijer J.P., Buick R., Gull M., Atlas Z. (2013). Evidence for reactive reduced phosphorus species in the early Archean Ocean. Proc. Natl. Acad. Sci. USA.

[21] Gull M., Mojica M.A., Fernández F.M., Gaul D.A., Orlando T.M., Liotta C.L., Pasek M.A. (2015). Nucleoside phosphorylation by the mineral schreibersite. Sci. Rep..

[22] La Cruz N.L., Qasim D., Abbott-Lyon H., Pirim C., McKee A.D., Orlando T., Gull M., Lindsay D., Pasek M.A. (2016). The evolution of the surface of the mineral schreibersite in prebiotic chemistry. Phys. Chem. Chem. Phys..

[23] Simonson B.M., Davies D., Wallace M., Reeves S., Hassler S.W. (1998). Iridium anomaly but no shocked quartz from Late Archean microkrystite layer: Oceanic impact ejecta?. Geology.

[24] Bryant D.E., Greenfield D., Walshaw R.D., Evans S.M., Nimmo A.E., Smith C.L., Wang L., Pasek M.A., Kee T.P. (2009). Electrochemical studies of iron meteorites: Phosphorus redox chemistry on the early Earth. Int. J. Astrobiol..

[25] Adande G.R., Woolf N.J., Ziurys L.M. (2013). Observations of interstellar formamide: Availability of a prebiotic precursor in the galactic habitable zone. Astrobiology.

[26] López-Sepulcre A., Balucani N., Ceccarelli C., Codella C., Dulieu F., Theulé P. (2019). Interstellar Formamide (NH_2_CHO), a Key Prebiotic Precursor. ACS Earth Space Chem..

[27] Gull M., Cafferty B.J., Hud N.V., Pasek M.A. (2017). Silicate-promoted phosphorylation of glycerol in non-aqueous solvents: A prebiotically plausible route to organophosphates. Life.

[28] Schoffstall A.M. (1976). Prebiotic phosphorylation of nucleosides in formamide. Orig. Life Evol. Biosph..

[29] Furukawa Y., Kim H.J., Hutter D., Benner S.A. (2015). Abiotic regioselective phosphorylation of adenosine with borate in formamide. Astrobiology.

[30] Schoffstall A.M., Barto R.J., Ramos D.L. (1982). Nucleoside and deoxynucleoside phosphorylation in formamide solutions. Orig. Life Evol. Biosph..

[31] Costanzo G., Saladino R., Crestini C., Ciciriello F., Di Mauro E. (2007). Nucleoside phosphorylation by phosphate minerals. J. Biol. Chem..

[32] Pasek M.A., Omran A., Feng T., Gull M., Lang C., Abbatiello J., Garong L., Johnston R., Ryan J., Abbott-Lyon H. (2022). Serpentinization as a route to liberating phosphorus on habitable worlds. Geochim. Cosmochim. Acta.

[33] Pasek M.A. (2018). Phosphorus NMR of Natural Samples.

[34] Gulick A. (1955). Phosphorus as a factor in the origin of life. Am. Sci..

[35] Schwartz A.W., Van der Veen M. (1973). Synthesis of hypophosphate by ultraviolet irradiation of phosphite solutions. Inorg. Nucl. Chem. Lett..

[36] Poulton S.W., Canfield D.E. (2011). Ferruginous Conditions: A Dominant Feature of the Ocean through Earth’s History. Elements.

[37] Guilbaud R., Poulton S., Butterfield N., Zhu M., Sheields-Zhou G.A. (2015). A global transition to ferruginous conditions in the early Neoproterozoic oceans. Nat. Geosci..

[38] Pasek M.A., Kee T.P., Bryant D.E., Pavlov A.A., Lunine J.I. (2008). Production of potentially prebiotic condensed phosphates by phosphorus redox chemistry. Angew. Chem. Int. Engl..

[39] Herschy B., Chang S.J., Blake R., Lepland A., Abbott-Lyon H., Sampson J., Atlas Z., Kee T.P., Pasek M.A. (2018). Archean phosphorus liberation induced by iron redox geochemistry. Nat. Commun..

[40] Ingalls M., Grotzinger J.P., Present T., Rasmussen B., Fischer W.W. (2022). Carbonate-associated phosphate (CAP) indicates elevated phosphate availability in Neoarchean shallow marine environments. Geophys. Res. Lett..

[41] Planavsky N.J., Rouxel O.J., Bekker A., Lalonde S.V., Konhauser K.O., Reinhard C.T., Lyons T.W. (2010). The evolution of the marine phosphate reservoir. Nature.

[42] Poulton S.W., Canfield D.E. (2006). Co-diagenesis of iron and phosphorus in hydrothermal sediments from the southern east Pacific Rise: Implications for the evaluation of paleoseawater phosphate concentrations. Geochim. Cosmochim. Acta.

[43] Halevy I., Bachan A. (2017). The geologic history of seawater pH. Science.

[44] Macia E. (2005). The role of phosphorus in chemical evolution. Chem. Soc. Rev..

[45] Pasek M.A., Lauretta D.S. (2008). Extraterrestrial flux of potentially prebiotic C, N, and P to the early Earth. Orig. Life Evol. Biosph..

[46] Ritson D.J., Mojzsis S.J., Sutherland J. (2020). Supply of phosphate to early Earth by photogeochemistry after meteoritic weathering. Nat. Geosci..

[47] Gull M. (2014). Prebiotic phosphorylation reactions on the early Earth. Challenges.

